# Surgical Management of Giant Genital* Condyloma Acuminata* by Using Double Keystone Flaps

**DOI:** 10.1155/2016/4347821

**Published:** 2016-11-16

**Authors:** Peri Eriad Yunir, Chaidir A. Mochtar, Agus Rizal A. H. Hamid, Chaula L. Sukasah, Rainy Umbas

**Affiliations:** ^1^Department of Urology, Faculty of Medicine, Universitas Indonesia, Cipto Mangunkusumo National Referral Hospital, Jakarta, Indonesia; ^2^Department of Surgery, Division of Plastic Surgery, Faculty of Medicine, Universitas Indonesia, Cipto Mangunkusumo National Referral Hospital, Jakarta, Indonesia

## Abstract

*Condyloma acuminata* in the external genitalia (genital warts) is a sexually transmitted disease that is often caused by human papillomavirus (HPV). We report a case of giant genital* condyloma acuminata* in a 35-year-old male patient with HIV comorbidity treated by wide surgical excision. Excision defect was covered with split thickness skin graft (STSG) and double keystone flaps. There was no complication after surgery. Ten months following surgery, there was no new* condyloma* lesion and the patient had normal voiding and erectile functions.

## 1. Introduction

External genitalia wart, also known as* condyloma acuminata*, is a common form of sexually transmitted disease. It is caused by human papillomavirus (HPV) [1, 2]. HPV infects the squamous epithelium and more commonly occurs on the external genitalia. Many treatment options (topical, systemic, or surgical) can be used for genital warts, but there has been no single specific and satisfying therapy [3–5].

## 2. Case Illustration

A 35-year-old man came to our clinic with painless cauliflower lumps on the external genitalia that appeared 1 year ago. There was no voiding problem. He had been treated with 90% trichloroacetic acid (TCA) and sodium fusidic ointment but the lumps remained enlarged. He had HIV and was being treated with antiretroviral (ARV) drugs.

**Figure 1:**
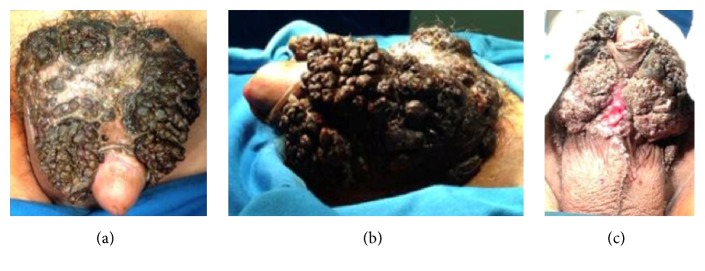
*Condyloma acuminata's* clinical manifestation. (a) Dorsal; (b) lateral; (c) ventral.

On physical examination, we found solid cauliflower-shaped (*condylomatosis*) lumps, covering almost the entire shaft of the penis, the suprapubic area, and the scrotum, with a size of ±9 × 8 × 4 cm (Figure 1). The urethral meatus was normal. Incisional biopsy of the lumps on the shaft of the penis had been performed with histopathological result of* condyloma acuminata*.

**Figure 2:**
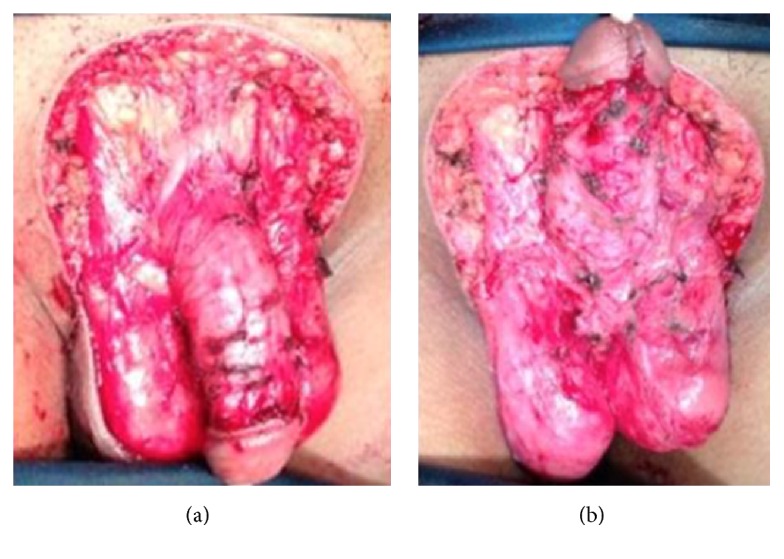
Wide excision on* condyloma acuminata*. (a) Dorsal; (b) ventral.

Degloving of the penis and wide excision with 1 cm margin were performed. The whole mass can be removed from the penile shaft (without infiltration to the corpus cavernosum and spongiosum) and subcutaneous tissues of the suprapubic area and scrotum (Figure 2). Frozen section revealed no tumour at all incision margins and excision bases.

**Figure 3:**
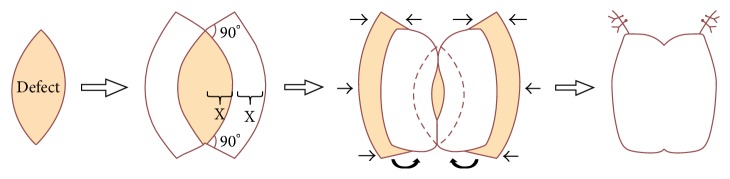
Double keystone flaps (modification from Bhat [6]).

**Figure 4:**
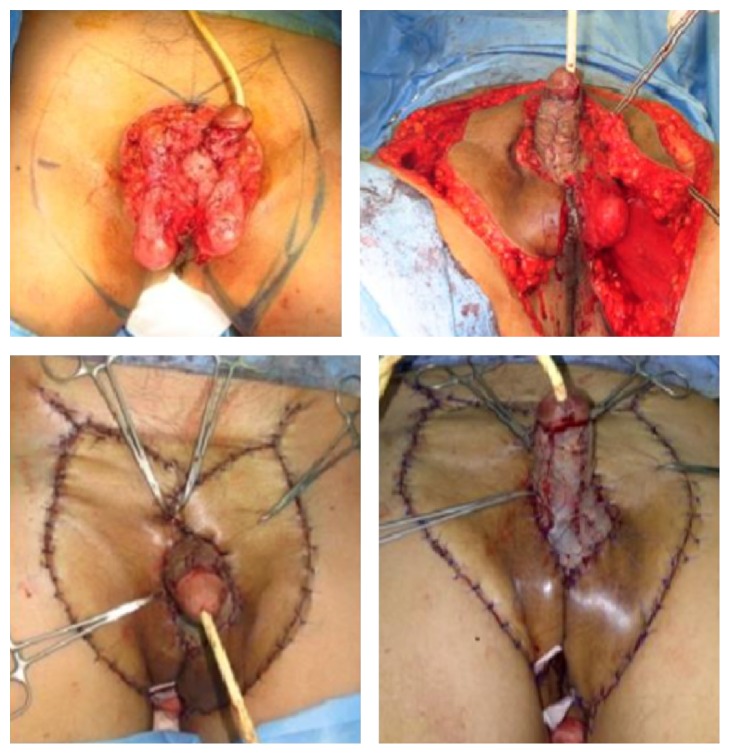
Excision defects were closed by STSG and double keystone flaps.

Subsequently, split thickness skin graft (STSG) had been performed to close the defect in the shaft of the penis and double keystone flaps had been used for scrotum and suprapubic defects. Double keystone flaps were performed by incision of the abdominal wall at each lateral aspect of the defect until the scarpa fascia with 1 : 1 ratio of excisional defect and flap width. One-third inferior part of double keystone flaps was undermined and rotated medially to close the scrotal defect. Flaps were elevated and attached to each other, closing the suprapubic defect (Figures 3 and 4).

**Figure 5:**
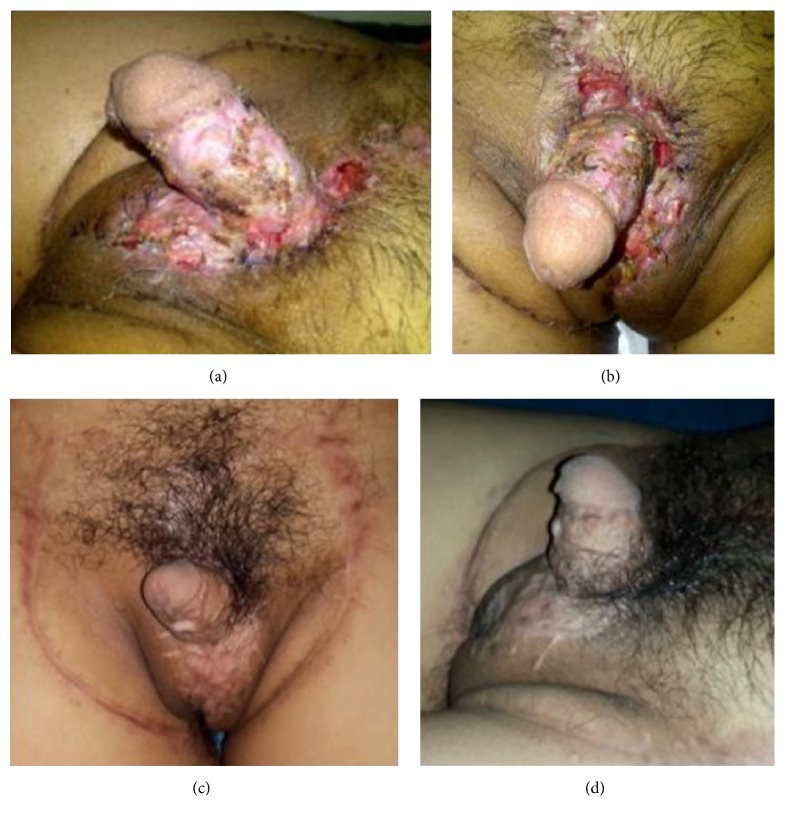
One-month follow-up (a, b) and 4 months (c, d) after surgery.

The histopathological result revealed* condyloma acuminata* with no evidence of malignancy. The surgical procedure was uneventful without complication after surgery. Ten months following surgery, there was no new* condyloma* lesion and the patient had normal voiding and erectile functions (Figure 5).

## 3. Discussion

Genital warts can manifest themselves as solitary or clustered lesion that may be flat, like a dome, keratotic, cauliflower, or pedunculated in shape and white, pink, purple, red, or brown in colour [7–9]. Warts can grow on the anus, pubis, and oral cavity besides external genitalia [2, 7]. They are usually asymptomatic but can be very painful and itchy and can cause bleeding and organ function impairment [4, 10]. The patient in this case had painless external* condyloma acuminata* on the external genitalia with cauliflower shape and was being treated with ARV for HIV infection.

Genital warts can be accurately diagnosed with a careful clinical history and physical examination. Biopsy is performed if the warts do not respond to therapy, especially when there is suspicion toward malignancy [3, 7, 9]. Identification of HPV type is not recommended for the diagnosis or management of genital warts [4, 5, 7, 11].

The main goal of treatment is to eliminate genital warts that may cause physical symptoms or emotional distress [4]. Current available treatments may or may not be able to cure the underlying HPV infection. HPV infection can still persist, even though the treatment can cure the genital warts [4]. If left untreated, genital warts can regress spontaneously, remain unchanged, or even increase in numbers, and they rarely become malignant [12–14].

Selection of therapy depends on the physical and psychosocial condition besides the availability of medical facilities. Size, anatomical location, and the amount and character of genital warts, as well as the presence of comorbidity such as pregnancy and immune system deficiency, will affect the therapy selection [4]. The treatments can be classified into patient-applied and provider-prescribed/applied treatments, which can be done by topical, systemic, or surgical approaches [2, 4, 15]. The patient in this case had been previously treated with topical therapy for 3 months. Considering the quite large size of* condyloma acuminata* and the no response to topical treatment, further management was done by surgery.

Wide excision of the mass was performed. Skin defect was closed by STSG for the penile shaft and double keystone flaps for the suprapubic area and scrotum. Another technique is to interdigitate the quadrangular shaped keystones pairs in a yin yang manner to decrease the tension of the flap for closing perineal or gluteal region defect. Keystone flap was developed by Felix Behan in 1995. Its concept was first published in 2003. Since that time, it has emerged for the locoregional fasciocutaneous reconstruction technique in numerous body regions. This technique was selected because it provides a single reliable flap that is easy to design, elevate, and offer rapid fasciocutaneous closure. The other advantages of the keystone flap are the good vascular supply, reliable healing, short operative time, less postsurgical care, minimal patient morbidity, relatively pain-free surgery, good aesthetic outcome, and cost-effective wound closure compared to other approaches [16]. Numerous studies showed its success for various defects at facial, trunk, and extremities regions with primary wound healing and satisfactory result, even in a coloured-skin population [6, 17, 18]. It also can be used in patients with full-thickness burns defect and a previous history of radiotherapy [19, 20].

Ten months after excision, no new* condyloma acuminata* lesion was found in the former site. There were no complaints in voiding and erectile function. Aesthetically, the patient was satisfied with the result of surgery. HIV therapy was continued with no further therapy for genital warts after the surgery.
